# Evaluating alternatives to the Widal test for typhoid fever diagnosis in developing countries: A targeted literature review

**DOI:** 10.4102/jphia.v16i1.700

**Published:** 2025-02-14

**Authors:** Bereket A. Tegene, Meron T. Eshetie

**Affiliations:** 1 School of Medicine, College of Medicine and Health Sciences, Hawassa University, Hawassa, Ethiopia; 2 School of Medicine, University of Gondar, Gondar, Ethiopia

**Keywords:** typhoid fever, Widal test, rapid diagnostic tests, sensitivity, specificity, low- and middle-income countries

## Abstract

**Background:**

Typhoid disease, caused by *Salmonella typhi*, is prevalent in developing countries with poor sanitation. The Widal test, despite its century-old history, has drawbacks in diagnostic capacity because of inherent characteristics, cross-reactivity and repeated exposure to the pathogen in endemic regions.

**Aim:**

The study evaluates the utility of the Widal test for the diagnosis of typhoid infections and emphasises the need for a better diagnostic modality in endemic regions.

**Setting:**

The study included research conducted in developing countries where typhoid fever is endemic.

**Method:**

A targeted literature review was conducted utilising the MEDLINE and Embase databases on 19 October 2022, encompassing publications from the preceding 10 years. Manual searches were executed using Google Scholar and the Google Search Engine on 02 November 2022. The initial search yielded 657 articles, of which 20 met the inclusion criteria and were subsequently incorporated into the final review.

**Results:**

The mean sensitivity, specificity, positive predictive value and negative predictive value of the Widal test in this study were 62.94 ± 17.83 (95% confidence interval [CI]: 49.23–76.64); 73.31 ± 18.75 (95% CI: 58.89–87.73); 58.85 ± 40.07 (95% CI: 16.80–100.90) and 75.96 ± 25.93 (95% CI: 46.08–100.45), respectively.

**Conclusion:**

The different studies in this review have shown that the Widal test performs poorly in identifying typhoid infections compared to other rapid diagnostic tests (RDTs). In addition, the current alternative RDTs are not accurate enough to reliably identify or rule out typhoid infection.

**Contributions:**

A shift in diagnostic approach for typhoid fever in developing countries is required and an accurate and feasible point-of-care test is urgently needed.

## Introduction

Typhoid fever is one of the infectious diseases which is caused by the bacteria *Salmonella typhi*.^1^ It remains one of the major health problems, causing significant morbidity and mortality in the developing world, where poor sanitation, inadequate water supply and ineffective sewage disposal systems create fertile ground for the feco-oral transmission of bacterial transmission. The total number of cases of typhoid fever reported in 2017 were 10.9 million.^2,3^ This disease is characterised by a broad spectrum of clinical manifestations ranging from being asymptomatic to having systemic manifestations such as fever, malaise and headache including life-threatening complications such as ileal ulceration, perforation and haemorrhage.^4^

It is estimated that every year 12–16 million cases of typhoid illness and 77 000–219 000 deaths occur throughout the world and the majority of the disease burdened with significant morbidity and mortality occurs in low- and middle-income countries (LMICs) mainly in South Asia, Southeast Asia and most African countries.^3^ However, an accurate estimation of the disease burden is limited by the overlap of clinical features of enteric fever with other febrile illnesses and the absence of tests with high diagnostic accuracy in endemic regions.^5,6^

The diagnosis of typhoid fever can be made using clinical signs and symptoms such as serological markers; blood, bone marrow and stool culture; and antigen detection and deoxyribonucleic acid (DNA) amplification.^7^ Definitive diagnosis of typhoid fever can be carried out by isolating *Salmonella typhi* from blood or bone marrow culture.^8^ Blood culture is regarded as the gold standard test with a diagnostic accuracy of 70% – 75% in the first week of illness.^9^ Blood, bone marrow and stool culture are the most reliable diagnostic methods, but they are expensive to perform.^7^

The Widal test was developed a century ago and remains widely used in many developing countries despite its drawbacks. Its limitations in diagnostic accuracy, including cross-reactivity with other infections and the effect of repeated exposure to the etiologic agent, affect its ability to correctly identify infections in endemic regions.^10^ Despite this, the Widal test has been used in the developing part of the world as it is relatively cheaper, easily performed and requires minimal training and laboratory resources.^7^ This has its own contribution in compromising the quality of health care services delivered possibly by affecting proper patient diagnosis and management, leading to inappropriate use of antibiotics and antimicrobial resistance. This in turn leads to poor patient satisfaction with overwhelmed health facilities because of frequent visits of patients for not showing improvement in their clinical condition. Other possible point-of-care tests include Typhidot, TUBEX, Test-it Typhoid and the Typhoid-Paratyphoid diagnostic assay (TPTest) but these are also criticised for having low sensitivity and specificity resulting in antibiotic overuse contributing to the emergence and spread of multidrug-resistant strains.^11^ Most importantly, the current diagnostic approach for typhoid in LMIC settings is not reliant on evidence-based medical practice.

The objective of this study is to assess the sensitivity, specificity, positive predictive value (PPV) and negative predictive value (NPV) of the Widal test in correctly identifying typhoid infection and suggest alternative diagnostic modalities aiming to emphasise the need for a shift in the use of the commonly used Widal test for the diagnosis of typhoid infections in developing countries.

## Methods

This targeted literature review (TLR) was performed in accordance with a pre-specified protocol. This involved searching electronic databases of the past 10 years, manual hand searching of Google and Google Scholar, manual hand searching of the WHO (World Health Organization) website, and manual hand searching of the bibliographies of any relevant systematic literature reviews (SLRs) and (network) meta-analyses ([N]MAs) identified during the review. MEDLINE (MEDLINE In-Process, MEDLINE Daily and MEDLINE Epub Ahead of Print) and Embase were searched simultaneously via the Ovid Scientific Platform (SP) platform on 19 October 2022. Google Scholar and Google Search Engine were manually searched on 02 November 2022 to identify any additional relevant publications for inclusion. The search strategies, including all search terms that were used in the selected databases, are provided in Online Appendix 1 Table S1–S2. The search terms mainly included terms related with typhoid disease (typhoid fever or typhoid illness) and diagnosis (sensitivity and specificity). Searches were limited to studies done in English in developing countries. The criteria in Table 1 were used to make sure that the included studies are relevant enough to meet the aim and objectives of this review. To assist the reviewer while screening articles, eligibility flow charts were developed and used based on the criteria for the title or abstract and full-text review stage (see Online Appendix 1 Figure S1–S2).

**TABLE 1:** Eligibility criteria for inclusion in the targeted literature review.

Domain	Inclusion	Exclusion
Population	All population in the LMICs who have been diagnosed as having typhoid disease	In vitro/preclinical/animal studies
Intervention	Widal test	Tests which are not used for the diagnosis of typhoid fever
Comparator	All other tests used for the diagnosis of typhoid disease (blood culture, bone marrow, PCR, etc.)	Studies not investigating a relevant intervention and/or comparator
Outcomes	Efficacy outcomes, including but not limited to: □ Sensitivity and specificity □ PPV □ NPV	Economic outcomes Epidemiological outcomes Studies not reporting any outcomes listed of relevance separately for the population of interest
Study design	Any primary research design, including randomised controlled trials, non-randomised interventional studies, observational studies (prospective and retrospective) or peer-reviewed articles reporting original research Guidelines or guidance documents	Case reports/case studies Comments or editorials Economic Evaluations Non-systematic or narrative reviews
Settings	LMICs	Studies carried out in the developed world
Language	Publication in the English language	Studies published other than English language
Other considerations	Date of publication (2012−2022)	Studies published before 2012

LMICs, low- and middle-income countries; PPV, positive predictive value; NPV, negative predictive value; PCR, polymerase chain reaction.

Each title and/or abstract was reviewed against the eligibility criteria by a single reviewer. Where the applicability of the inclusion criteria was unclear, the article was included at this stage to ensure that all potentially relevant studies were captured. A search for freely available full-text articles required for the TLR was conducted, and those which were found to be paywalled were purchased, where appropriate. Each full-text article was reviewed against the eligibility criteria by a single reviewer, with a second reviewer reviewing all articles included at this stage. A third reviewer was used to resolve issues between reviews when conflicts arise in applying criteria.

Basic information about the final included studies was extracted into a predesigned data extraction grid that includes diagnostic performance of the various tests used for diagnosis of typhoid fever, outcomes and given recommendations (see Online Appendix 1 Table S4).

### Ethical considerations

This article followed all ethical standards for research without direct contact with human or animal subjects.

## Results

**FIGURE 1:**
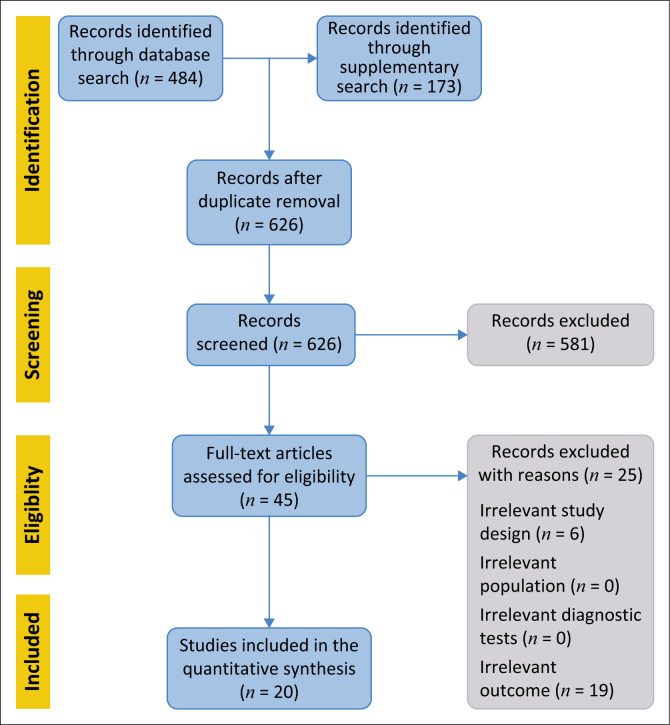
Preferred reporting items for systematic reviews and meta-analyses flow diagram.

A total of 657 unique articles were identified from the electronic database searches and after removing duplicates, 626 records were reviewed at the title/abstract review stage. After title/abstract review, 45 were deemed potentially relevant and were reviewed at the full-text stage, with 20 articles meeting the inclusion criteria. A Preferred Reporting Items for Systematic Reviews and Meta-Analyses (PRISMA) diagram outlining the flow of records through the TLR is presented in Figure 1.

### Summary of included studies

Overall, 20 studies were included in the review which focusses on the diagnostic accuracy of the Widal test and other alternative tests in comparison with the gold standard in different settings. Five studies were conducted in India, three studies were conducted in Nigeria and the remaining 12 were conducted in Bangladesh, Ghana, Ethiopia, Libya, Cameroon, Pakistan, Kenya, Nepal and Vietnam. The review included six cross-sectional studies, four case-control studies and a range of other study types including a supplemental article, Delphi survey, observational study, chart reviews and conference papers. The included studies were English articles published from 2012 to 2022. We used the Critical Appraisal Skills Programme (CASP) framework for the quality appraisal of the included studies.

### Sensitivity, specificity, positive predictive value and negative predictive value

Sensitivity is the ability of the test to correctly identify patients with a disease. Specificity is the ability of a test to correctly identify patients without a disease, while specificity is the ability of a test to correctly identify people without the disease.^12^ Positive predictive value is the probability of those testing positive by the test that is truly infected, whereas NPV is the probability of those testing negative by the test that is truly uninfected.^13^

To distinguish the diagnostic accuracy of the Widal test, the average of the sensitivity and specificity reported by the included studies was calculated and used to help draw conclusions despite the heterogenicity of the evidence found across different study types and patient populations.

### The utility of Widal test

Among the included studies, blood culture was used as a gold standard in all of the comparative studies. Across the included studies, the sensitivity of the Widal test ranged from 34.7% to 94.4% and the mean sensitivity was 62.94 ± 17.83 (95% confidence interval [CI]: 49.23–76.64). The highest sensitivity was recorded in a study carried out in Cameroon in 2021 while it was less than 80% in the remaining studies.^14^

The mean specificity of the Widal test was 73.31 ± 18.75 (95% CI: 58.89–87.73) and ranged from 48.35% to 100%; only in two studies, conducted in Nigeria and Vietnam,^15,16^ were the specificity found to be above 90%. The PPV of the Widal test was 58.85 ± 40.07 (95% CI: 16.80–100.90) and ranged from 5.7% to 100% and the NPV was 75.96 ± 25.93 (95% CI: 46.08–100.45).

### Alternative diagnostic tests

Among the included studies, the sensitivity of the Typhidot test ranged from 26.7% to 100.0% with a mean sensitivity of 78.36 ± 25.41 (95% CI: 54.86–101.86) while its specificity ranged from 61.7% to 97.4% with mean specificity of 83.65 ± 12.55 (95% CI: 72.04–95.25) while the PPV of the Typhidot test ranged from 7.4% to 98.18% with mean PPV of 64.52 ± 39.54 (95% CI: 23.02–106.01) and the mean NPV was 87.63 ± 15.08 (95% CI: 71.80–103.46).

In a study conducted in India, the Enteroscreen-immunoglobulin M (Igm) test showed a sensitivity, specificity, PPV and NPV of 88.13%, 87.83%, 92.03% and 82.27%, respectively while the Dot Blot Assay IgM + immunoglobulin G (IgG) has comparative values of 51.92%, 74.57%, 78.26% and 46.80%, respectively.^17^ The standard diagnostics (SD) Bioline *Salmonella typhi* IgM and IgG Fast have a low sensitivity of 21.6% and 9.1% while with high specificity of 100% and 99.6%. Typhidot Rapid IgM and IgG combo test have a relatively low sensitivity of 46.2% and 11.4% despite higher specificity of 82.8% and 98.9%.

The study conducted in Kenya and Pakistan^18^ showed that Enter check whole blood (WB) has a sensitivity and specificity of 72.7% and 86.5%; Test-it typhoid IgM has a sensitivity of 63.6% and specificity of 95.1%. Typhoid IgG and IgM Combo Rapid Test CE (CTK) have a sensitivity of 78.8% and 49.6% and a specificity of 59.2% and 78.7%, respectively. Typhoid IgG Rapid Test Cassette has a low sensitivity of 31.8% and a high specificity of 91.8% while Diaquick s.typhi/paratyphi Ag cassette had 0% sensitivity and 100% specificity. Tubex TF has shown a sensitivity of 60.6% and specificity of 94% in the same study while the study carried out in Vietnam showed a sensitivity and specificity of 78% and 94% respectively.^16,18^ Multi-Test Dip-S-Ticks for Serotype Typhi have shown a sensitivity of 89% and specificity of 50%.^16^

## Discussion

**FIGURE 2:**
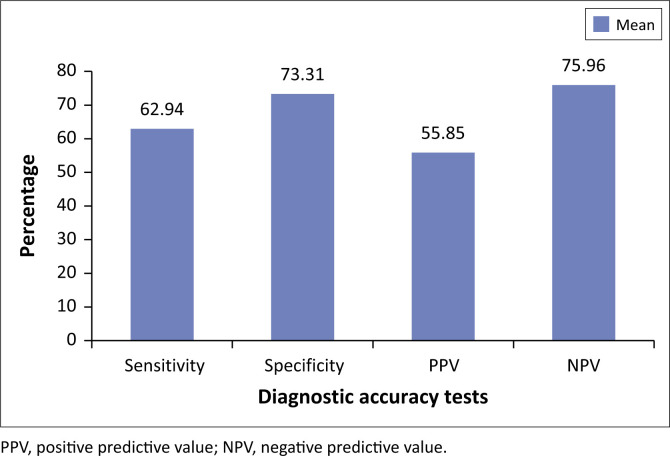
Diagnostic accuracy of the Widal test.

This study showed the Widal test performed poorly in different studies conducted in developing countries with mean sensitivity and specificity of 62.9% and 73.3%, respectively (Figure 2). This indicates that nearly one-third of patients who are truly infected by typhoid fever are missed while using this test, and almost one-fourth of patients who do not have the infection are considered to be infected. The mean PPV of the Widal test was found to be 64.52% which implies there is only a 65% chance of being infected despite getting a positive test result. The inability of the test to properly identify infected individuals and rule out those uninfected leads to misdiagnosis and thus inappropriate patient management. This further increases morbidity and mortality imposed by typhoid disease in the developing world. Furthermore, it will predispose patients to unnecessary health care expenditure which contributes to worsening poverty in endemic regions. Most importantly, the use of antibiotics for those uninfected individuals because of the misleading nature of the test leads to irrational use of medicine in routine clinical practice thus increasing the chance of antimicrobial resistance. This is evidenced by the fact that new strains of *S. typhi* which are resistant to three first-line agents are now prevalent in parts of Asia and Africa.^19^

One of the reasons behind the low performance of the Widal test is the lack of consistent cut-off values across laboratories making patient diagnosis and management more difficult.^13^ In endemic regions, a significant proportion of healthy individuals exhibit seropositivity against O and H antigens for *S. typhi* or *S. paratyphi* because of the high prevalence of salmonella infections. The presence of antigens shared with other salmonella serotypes, as well as cross-reactivity with some Enterobacteriaceae and parasites like *Plasmodium falciparum*, further complicates accurate diagnosis.^14,20^ This misleads the interpretation of the Widal test giving false positive results in patients without actual typhoid infections who visit health facilities for other similar complaints.

The poor diagnostic value of the Widal test for typhoid fever has led researchers to conduct comparative studies in different study settings in search of better alternative tests. A comparative study conducted in India has also shown that the Typhidot has a higher sensitivity and specificity of 92% and 83%, respectively, while the Widal test had a sensitivity and specificity of 53% and 83%, respectively. This is consistent with another study conducted in Pakistan where Typhidot performed better than Widal with a sensitivity and specificity of 97.36% and 63.15%, respectively.^8^

In this review, the mean sensitivity and specificity of the Typhidot, a test which detects IgM and IgG antibodies against salmonella bacteria, is 78.36% and 83.65% which has relatively better diagnostic performance than the Widal test. This signifies that the Typhidot is superior to the routinely used Widal test and could be used as an alternative test.^21,22^ On the other hand, a multicentre diagnostic study conducted in Kenya and Pakistan evaluated the sensitivity and specificity of the current commercially available rapid diagnostic tests (RDTs) for typhoid diagnosis and reported that they fell below the required level for accurate diagnosis. However, the Enterocheck WB showed the best performance with a sensitivity of 72.7% and specificity of 86.5% compared to the other 12 diagnostic tests including the Widal test.^18^ This aligns with other comparative studies carried out in India where Enterocheck WB has better diagnostic accuracy with 85.5% sensitivity, 88.6% specificity, 97.7% PPV and 51.1% NPV.^23^ The remaining tests which were included in this study have shown poor diagnostic advantage.

Various studies conducted in different countries have indicated that the current RDTs used for diagnosing typhoid fever in low-resource settings are not accurate enough to reliably identify or rule out the infection, leading to improper patient management. World Health Organization has suggested a test which is used for the diagnosis of typhoid fever in endemic resource-limited settings should be sensitive, specific, affordable, user-friendly, rapid, robust and deliverable.^11^

A study conducted to determine the characteristics of an ideal point-of-care test for typhoid diagnosis suggested that the point-of-care (POC) test should have a sensitivity of ≥ 90%, a specificity of ≥ 95% and an end-user cost of less than $3.00. The tests developed so far do not fulfil this requirement, and this highlights the need for an urgent better diagnostic modality for typhoid infections that can be used in low-resource settings.^11^ So far no test for the diagnosis of typhoid infection has been developed to meet this requirement.

This study has its limitations, as not all included studies were comparative, resulting in a lack of uniformity among the different studies. Furthermore, most of the comparative studies used blood culture as a gold standard reference, which has limited sensitivity. Additionally, the calculated mean sensitivity, specificity, PPV and NPV for Widal and Typhidot were derived from various study types and patient populations, leading to heterogeneity in the evidence.

## Conclusion

The findings of this TLR indicate that the diagnostic accuracy of the Widal test is poor across different studies, as it shows cross-interference with other febrile illnesses. Therefore, it should not be used for diagnosing or guiding the management of patients presenting with non-specific signs and symptoms of typhoid fever in endemic regions.

The use of relatively better alternative diagnostic tests, such as Typhidot and Enterocheck, should be encouraged until a new POC test feasible for low-resource settings is developed. In diagnosing typhoid fever, where no single perfect test exists, reliance on a single test should be avoided. Instead, the use of a composite reference standard (CRS) could be considered as an alternative.

It is urgently needed to develop a new POC test with high diagnostic accuracy which is feasible to be used in LMIC settings. Meanwhile health policies and regulations that promotes capacity building in using relatively better diagnostic tools like alternative RDTs and blood culture should be implemented. Therefore, collaboration among research institutions, companies producing diagnostic tools and other stakeholders is crucial in producing new and updated POC tests that can be used in typhoid endemic regions.
